# Human Thanatomicrobiome Succession and Time Since Death

**DOI:** 10.1038/srep29598

**Published:** 2016-07-14

**Authors:** Gulnaz T. Javan, Sheree J. Finley, Ismail Can, Jeremy E. Wilkinson, J. Delton Hanson, Aaron M. Tarone

**Affiliations:** 1 Forensic Science Program, Physical Sciences Department, Alabama State University, Montgomery, AL 36104 USA; 2 Ph.D. Program in Microbiology, Department of Biological Sciences, Alabama State University, Montgomery, AL 36104 USA; 3 Research and Testing Laboratory, Lubbock, TX 79407 USA; 4 Department of Entomology, Texas A&M University, College Station, TX, 77843 USA

## Abstract

The thanatomicrobiome (*thanatos*, Greek for death) is a relatively new term and is the study of the microbes colonizing the internal organs and orifices after death. Recent scientific breakthroughs in an initial study of the thanatomicrobiome have revealed that a majority of the microbes within the human body are the obligate anaerobes, *Clostridium spp*., in the internal postmortem microbial communities. We hypothesized that time-dependent changes in the thanatomicrobiome within internal organs can estimate the time of death as a human body decays. Here we report a cross-sectional study of the sampling of 27 human corpses from criminal cases with postmortem intervals between 3.5–240 hours. The impetus for examining microbial communities in different internal organs is to address the paucity of empirical data on thanatomicrobiomic succession caused by the limited access to these organs prior to death and a dearth of knowledge regarding the movement of microbes within remains. Our sequencing results of 16S rRNA gene amplicons of 27 postmortem samples from cadavers demonstrated statistically significant time-, organ-, and sex-dependent changes. These results suggest that comprehensive knowledge of the number and abundance of each organ’s signature microorganisms could be useful to forensic microbiologists as a new source of data for estimating postmortem interval.

Human Microbiome Project (HMP) metagenomic sequencing studies characterized the complexity of the human microbiome at numerous body sites, highlighting significant differences between microbial communities within and among individuals^1^. In a healthy adult, a ratio of 10:1 of the cells in a body are microbial^1^,^2^. Sender *et al*.^3^ have challenged this estimate and suggested that the ratio is much closer to 1:1. The thanatomicrobiome is the postmortem microbial community of the human body, which involves a successional process where trillions of microbes inhabit, proliferate, and die internally and externally throughout the dead body, resulting in temporal shifts in community composition over time^4^,^5^. Upon death, the host environment changes due to the decomposition of cells and subsequent release of cellular components to the surrounding tissues. There are several sequential events that occur after a person dies that lead to uninhibited reproduction of certain types of microbial cells and the quiescence or death of other cells^6^,^7^,^8^,^9^,^10^.

The study of the thanatomicrobiome of internal organs and blood is not directly influenced by the same environmental abiotic factors (i.e., pH and temperature) and biotic factors (i.e., insects and scavenger activities)^11^ that are encountered by the necrobiome^12^. Additionally, it is presumed that the thanatomicrobiome of certain organs is not immediately affected by gut-associated microorganisms that proliferate rapidly after human death^13^. It was once a long held belief that human internal organs were sterile in living hosts^14^,^15^. However, there is sufficient evidence demonstrated by HMP data that some internal organs (i.e., lungs and gut) provide distinct niches for commensal opportunistic and pathogenic microorganisms to colonize microbiomes^1^. The microbes discovered in the internal organs of cadavers could represent those directly associated with human decomposition. Tuomisto *et al*.^13^ demonstrated that internal organs such as the liver remain sterile up to five days after death.

As a human body decays, microbes proliferate in the blood, liver, spleen, heart and brain in a time-dependent manner; therefore, the relative abundances of microbes will vary by respective body organ and postmortem interval (PMI)^5^. In order to address the dearth of knowledge regarding the internal thanatomicrobiome, we describe here results of the largest study thus far of the postmortem microbial communities of internal organs. We hypothesized that as a human body decomposes, the thanatomicrobiome within internal organs shifts in microbial community structure as time progresses. To assess this hypothesis, we sampled 27 corpses with varying times of death (range 3.5–240 hours), across multiple causes of death, using 16S rRNA amplicon sequencing technologies to classify the microbial taxa associated with the organs of cadavers. Further, we demonstrated a significant correlation in the thanatomicrobiome signatures, which corresponded with PMI. Thus, we produced a microbial catalogue of the thanatomicrobiome, which may establish a useful forensic tool and provide constructive information to those who study human remains.

## Results

To investigate thanatomicrobiomic profiles generated by 16S rRNA gene amplicon sequencing, we selected and analyzed the internal organs (brain, heart, liver, and spleen), buccal cavities, and/or blood of 27 human corpses from actual criminal cases with postmortem intervals between 3.5–240 hrs were collected (Table S1). In total, we sequenced microbial DNA for 66 specimens from the 27 cadavers. The rarefaction curves demonstrated that when the number of sequence reads increased, the species richness rose considerably with each sample (Fig. S1). The alpha diversity demonstrated by the rarefaction curves showed that the tissues were sufficiently sequenced to observe all taxa. Most samples reached an asymptote, indicative of adequate reads for the comparison of the specimens.

**Figure 1:**
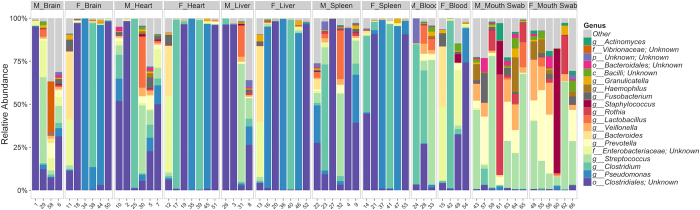
Relative abundance of the 20 most predominant genera in all samples.

Individual operational taxonomic units (OTUs) were examined for statistical significance, and the relative abundances of the 20 most predominant genera in all samples were determined (Fig. 1). The results showed that bacterial genera were similar among different organs within each sex, but were dissimilar between females and males. One exception to this trend is the buccal cavity, for which similar genera were seen for both sexes as shown in Fig. 1. With the exclusion of three mouth specimens, the remaining samples form a distinct cluster in the heatmap. Weighted UniFrac ADONIS on the mouth samples resulted in a highly non-significant difference among sexes (p = 0.923). The relative abundances of microbes in blood samples were similar in each specimen as time progressed (Fig. 1). The ANOVA, examining differences in Shannon diversity, elucidated significant differences among all organ types (p < 0.05) (Table 1). Brain, heart, liver, and spleen samples had similar relative abundances of the same bacterial taxa over time and sexes (Table 1).

**Figure 2:**
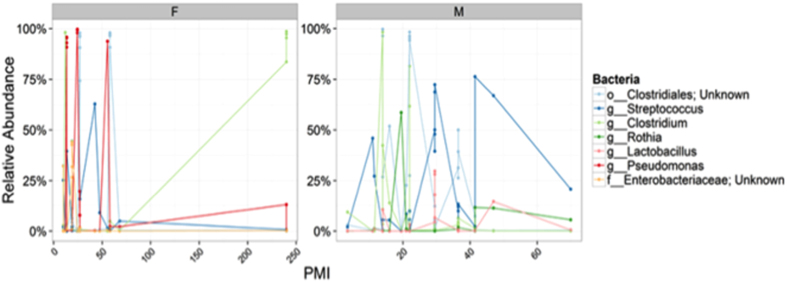
Relative abundance of the most predominant bacteria between females and males samples.

In Fig. 2, the body sites with significantly different α-diversity values between females and males are shown. There were significant differences between females and males (p < 0.001). Female cadavers had a high relative abundance of *Pseudomonas* and *Clostridiales*, whereas males had *Clostridium, Clostridiales,* and *Streptococcus* in high abundance with the lapse of time. Moreover, the most abundant genus in females was the aerobic, Gram-negative *Pseudomonas*. On the contrary, interestingly only males contained the facultative anaerobic, Gram-positive *Rothia*.

**Figure 3:**
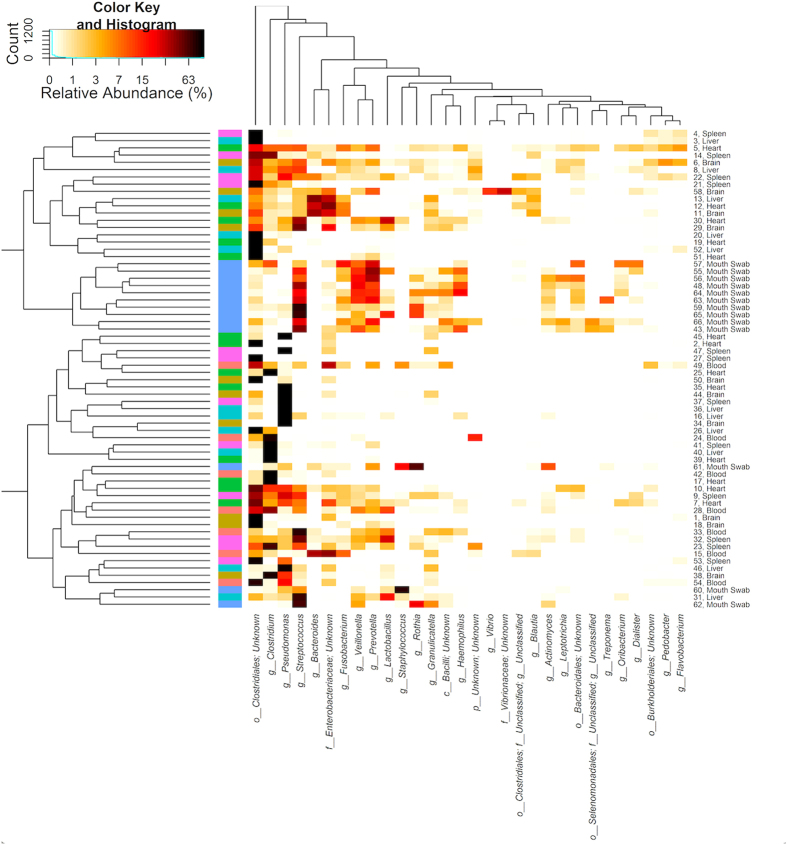
Heatmap to visualize the relative abundances of the 30 most predominant bacterial genera. Bacterial genera and samples were sorted based on Euclidean and unweighted UniFrac distances, respectively.

A heatmap was generated to visualize the relative abundances of the 30 most predominant bacterial genera (Fig. 3). The samples and bacterial genera were sorted according to Euclidean metrics and unweighted UniFrac distances, respectively. The heatmap showed similar relationships; the buccal cavity contained similar organisms (e.g., *Streptococcus, Viellonella, and Prevotella*), with all other organ types possessing a more similar community to one another than the buccal cavity.

The measures of microbial diversity were screened for differences using ADONIS testing based on UniFrac distances (Table 2). Significant differences (p < 0.05) were observed in internal organs, sex, and PMI for both unweighted and weighted UniFrac ADONIS tests.

**Figure 4:**
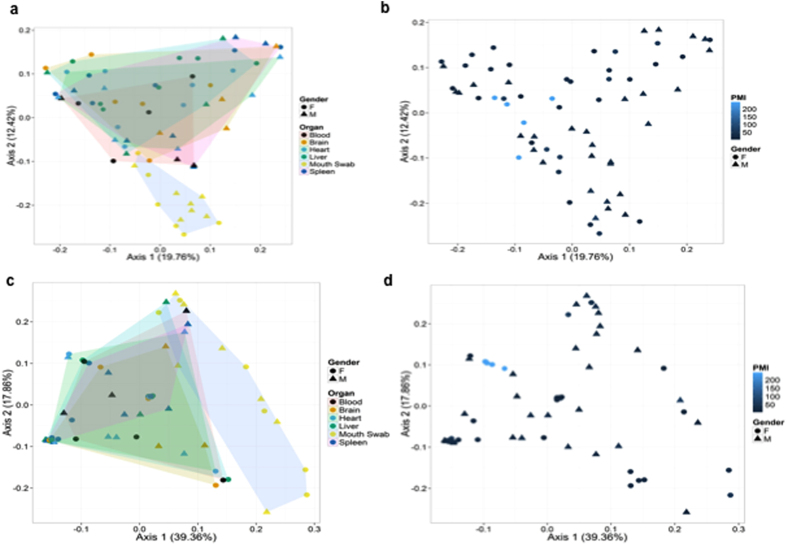
(**a)** Principal Coordinates Analysis (PCoA) of beta diversity based on unweighted Unifrac distances. (**b**) PCoA based on unweighted Unifrac distances, with PMI illustrated. (**c)** PCoA based on weighted Unifrac distances and (**d**) weighted Unifrac distances with PMI illustrated.

Principal Coordinates Analysis (PCoA) based on unweighted and weighted Unifrac distances was determined for all samples (Fig. 4). The PCoA based on unweighted UniFrac distances revealed that buccal cavity samples formed a distinct group from the other specimen types (Fig. 4a). The PCoA for female samples with a high PMI congregated close to 0 for both axes, as shown in Fig. 4b. As seen in the unweighted UniFrac distances PCoA, the same distinct buccal cavity group was depicted in the weighted PCoA (Fig. 4c). For the weighted UniFrac distance PCoA with PMI illustrated, the samples with a high PMI were located all in one area at around (0.1, −0.1). Once again, these were all female samples that had a high PMI (Fig. 4d).

**Figure 5:**
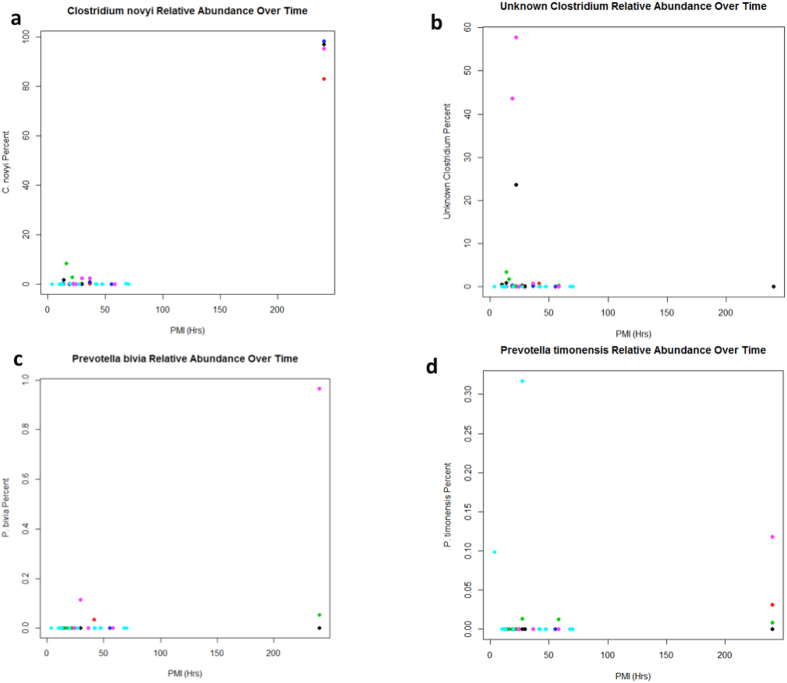
Taxa identified from a random forest analysis of the correlations between PMI and OUTs. Each organ is represented by a different color: Red = Brain, Green = Heart, Blue = Liver, Black = Blood, Cyan = Mouth, Pink = Spleen. The X-axis is PMI in hours, and the Y-axis represents the percentage of sequence reads attributed to that species in the sample. Results are presented for (**a**) *Clostridium novyi*, (**b**) an unknown *Clostridium*, (**c**) *Prevotella bivia*, and (**d**) *Prevotella timonensis*.

An analysis was performed to determine correlations between PMI families, genera, or species using random forest analysis (Fig. 5, Table S2)^15^,^16^,^17^,^18^. The results identified a list of candidate taxa that changed in abundance over time across sexes and sample types (Table S2). These analyses demonstrate an unknown *Clostridium sp*. (Fig. 5a), *Clostridium novyi* (Fig. 5b), *Prevotella bivia* (Fig. 5c), and *Prevotella timonensis* (Fig. 5d). For both genera, there were different species that increased in abundance early in decomposition that were different from genera that dominated at longer PMIs. Interestingly, several genera, such as *Clostridium* and *Prevotella*, possessed various species that were potentially predictive of different periods of decomposition. For instance, *C. novyi* was relatively more abundant at the latest PMI; yet an unknown *Clostridium* species was more abundant earlier in decomposition.

**Figure 6:**
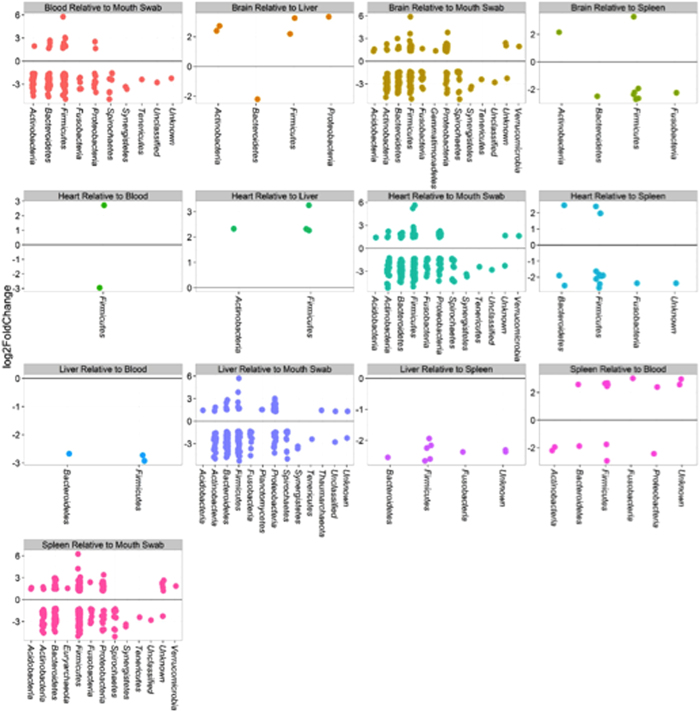
Results of comparisons to identify phyla with significantly different abundances between cadaver specimens. Samples with p values < 0.05 after Benjamini-Hochberg correction for multiple testing are shown.

The identities of the most abundant phyla were also determined to show the significantly different abundances between organs (Fig. 6). Surprisingly, Firmicutes (which includes *Clostridium*) is listed in all comparisons, signifying that it is likely a stable biomarker across thanatomicrobiome communities derived from different locations in the body. Samples with p values < 0.05 after Benjamini-Hochberg correction for multiple testing were included. The buccal cavity resulted in the most distinct profile with the greatest log2 fold changes representing the most bacterial phyla when compared to other organs. Remarkably, Firmicutes bacteria found in the buccal cavities were also detected in all internal organs and blood. While random forest were used to assess significant correlations between PMI, we also performed Firmicutes phylum analysis (Fig. 6) across sample types. Although phylum was not very predictive of PMI, note that genera from Firmicutes, (e.g. *Clostridium, Bacillus, Peptoniphilus, Blautia, Lactobacillus*), exhibited temporal changes. Also, *Clostridium, Finegoldia, Peptoniphilus* have higher importance scores than organ in the random forest analysis (i.e. in the top ten candidates).

## Discussion

We describe in the present study how we compiled the most comprehensive amplicon based sequencing survey to date for evaluating the human thanatomicrobiome of actual cadavers from criminal casework. Despite the abundance of microbial decomposers in cadavers, there is a paucity of details on the specific microorganisms involved in the decay of human internal organs. In healthy humans, most internal organs are thought to be sterile or mostly void of microorganisms due to constant surveillance by the immune system^14^,^19^. After death, the immune system is no longer functional, and microbial proliferation is facilitated by the nutrient-rich environment of the cadaver^5^,^11^,^17^,^18^,^20^.

Human decomposition is attributed primarily to bacteria; however, a review of scientific literature demonstrates a lack of comprehensive empirical knowledge about the identities of the bacteria proliferating in human internal organs after death. For example, Can *et al*.^5^ conducted a microbial survey of the thanatomicrobiome associated with internal organ tissues of eleven cadavers using culture-independent Roche 454 pyrosequencing methods. The results of the study showed that the obligate anaerobe, *Clostridium*, was found in cadavers of varying times of death, while the facultative anaerobe, *Lactobacillus*, was more abundant in cadavers with shorter intervals after death (i.e. 29.5 hrs versus 240 hrs)^5^. According to the Hyde *et al*. (2013 and 2015) exploratory analyses of bacteria present in the buccal cavity and rectal scrapings of corpses decomposing in natural settings, it was demonstrated that within a cadaver-sampling site, there was variation between the onset and end points of the bloat stage^21^,^22^.

An interesting finding of this study is the occurrence of a *Pseudomonas sp.* exclusively detected in female cadavers versus a *Rothia sp.* found in a male cadaver. *Pseudomonas* is an aerobic, Gram-negative bacillus that causes serious opportunistic infections in humans.

Several interesting features were identified in the random forest analysis (Fig. 5, Table S2). First, as the depth of taxonomic level in the analysis increased (going from kingdom to species), so did the reported percent variance explained. Family and genus level analyses explained approximately 21% of variance in models correlating PMI with the variables of interest, and species level analyses explained 65% (Table S2). Secondly, internal organ was consistently ranked as one of the top 14 most important factors in the models and sex was in the top 38.

Creating a catalogue of the discrete microbial contributors that govern the structural and dynamic changes of the human thanatomicrobiome is essential for ascertaining how human putrefaction occurs. Other research efforts have taken similar approaches to interrogating the microbiota of decay using human surrogates, such as mouse and swine^12^,^16^,^18^ and have determined that the abundance of certain bacteria (e.g., Firmicutes) at specific time points during decay is indicative of the time of death. For example, in Metcalf *et al*. (2014), a “microbial clock” was demonstrated in a mouse model and the PMI estimates corroborated the actual PMIs within approximately three days^16^. Similarly, the results of our study showed Firmicutes as a potential biomarker across thanatomicrobiome communities.

One particularly important area to evaluate is the fact that this study did not identify ambient temperature exposure as a significant contributor to differences in community structure. There could be error in the definition of those thermal exposures because the corpses were found in different locations at different temperatures^23^. Alternatively, the body may be sufficiently insulating internal organs from thermal variation so that other factors (e.g. sex) can exert more of an influence on the community.

The current study presents the potential for a novel approach to determine time of death using expertise in forensic genetics, DNA sequencing, and bioinformatics. To date, our study represents the largest catalogue of microbial diversity of internal components of the human thanatomicrobiome. Furthermore, this study has revealed new information on how microbial populations change in different internal specimens by time when a human dies, which is currently not well-established. Determining the time within a narrow window is of ultimate importance in criminal investigations and is consistent with the medicolegal assignment of providing objective, evidence-based knowledge and tools to meet the challenges of forensic genetics. This information is also useful for pathologists and other researchers that work with decomposing human and animal remains, as it provides information regarding the identity, location, and timeline of microorganism colonization that is likely to be encountered after death. Future directions will include studies to explain the occurrence of *Rothia*, a Gram-positive cocco-bacilli that causes a variety of serious infections, which was discovered in a male case that died from an accidental drug overdose.

### Human Postmortem Microbiome Project (HPMP)

The Human Postmortem Microbiome Project (HPMP) endeavors to cultivate data that represents an extensive resource cataloguing the abundance and variety of microorganisms involved in humans (and/or human surrogates) decomposition^4^. The role of the working group is to provide a framework for internal (thanatomicrobiome) and external (necrobiome and gravesoil) microbial communities. The primary objective of the HPMP is to improve the understanding and practical applications relevant to solving questions concerning manner of death and postmortem interval estimations. Further, this project will foster concerted efforts to validate and standardize protocols to establish a framework for use in forensic investigations.

## Methods

### Cadaver cases and sample collection

Sixty-six samples derived from blood, brain, buccal cavity, heart, liver, and spleen of 27 human corpses (15 males and 12 females) from criminal cases (homicide, suicide, and overdose) with postmortem intervals between 3.5–240 hrs were collected. The study was approved by Alabama State University Institutional Review Board (IRB) number 2016011. Cadavers from several Alabama and Florida morgues were employed to assemble the study cadavers. The methods were in accordance with the relevant guidelines and regulations regarding working with cadavers as established by Alabama State University IRB. Informed consent was obtained from next-of-kin relatives of the cases. PMIs were established from official Daily Crime Logs generated and certified by local police departments. Corpses were kept in the morgues at 1 °C until the time of tissue collection. The age, sex, ethnicity, manner of death, ambient temperature, and PMI at the time of sampling were documented for each corpse (Table S1). Microbial swab samples were taken from buccal cavities and blood where applicable. Sections of the internal organs were dissected using sterile scalpels and placed in polyethylene bags in an examination area at 20 °C ambient temperature. Blood samples were collected from the heart and femoral veins and placed in 10 ml BD vacutainer EDTA tubes (Becton Dickinson) and 200 μl were directly analyzed. Sterile cotton tip CultureSwab applicators (Becton Dickinson) were swabbed forcefully (vigorous rubbing) across all sides of the tissues samples. Specimens were placed in a freezer at −80 °C until further analysis.

### Thanatomicrobiome DNA extraction, PCR amplification, and MiSeq Data Analyses

Approximately 50 μl of blood or 10 mg of thawed organ tissues were removed using a sterile scalpel, and DNA was extracted using the phenol/chloroform method as previously described^5^. DNA extracts were analyzed by NanoDrop^TM^ 2000 (ThermoScientific) and 1% agarose gel electrophoresis (in TBE 0.5 X). 16S rRNA genes were amplified by universal primers for the V4 region (515F–806R) using the Qiagen HotStarTaq Master Mix (Qiagen Inc.). Microbial sequencing was performed on the MiSeq platform (Illumina)^24^,^25^. Samples were amplified for sequencing in a two-step process. Primers for the first step were constructed using 515F–806R with the Illumina i5 and i7 sequencing primers added to the 5′ end of each, respectively. Products from the first amplification were added to a second PCR step based on qualitatively determine concentrations (amplicons were run on a 2% ethidium gel, gel bands were scored, and a volume of products was added to the second PCR based on the scores). Primers for the second PCR step were designed using Illumina Nextera PCR primers with 8 bp dual indexes. Amplification products were visualized with eGels (Life Technologies). Products were then pooled equimolar and each pool was size selected in two rounds using Agencourt AMPure XP (BeckmanCoulter) in a 0.7 ratio for both rounds. Size selected pools were then quantified using the Qubit 2.0 fluorometer (Life Technologies) and loaded on an Illumina MiSeq 2 × 300 flow cell at 10 pM.

The sequence data were then analyzed at Research and Testing Laboratory using a standard microbial diversity analysis pipeline consisting of two major stages, the denoising and chimera detection stage and the microbial diversity analysis stage. During the denoising and chimera detection stage, denoising was performed using various techniques to remove short sequences, singleton sequences, and noisy reads. Next, chimera detection was performed to remove chimeric sequences. Lastly, the remaining sequences were then corrected base-by-base to remove noise from within each sequence. During the diversity analysis stage, each sample was run through an analysis pipeline to cluster the reads into OTUs which then went through taxonomic classification to identify the species level.

### Biostatistical Data Analyses

For a given sample, the OTUs for that sample was divided by the total number of reads. For a group of samples or all samples, the number of reads for the OTUs for all samples or a group of samples was divided by the total number of reads. Then the OTU relative abundances were sorted by abundance (high to low). Microbial diversity of the cadaver samples was examined from two perspectives. First, overall richness (i.e., number of distinct nucleic acid sequences present within the microbiome) was expressed as the number of OTUs and was quantified using the Chao1 richness estimator. Secondly, the overall microbial diversity, which was determined by both richness and evenness and the distribution of abundance among distinct taxa, was expressed as Shannon-Wiener Species Diversity. Measures of microbial diversity were screened for group (organ, manner of death, ethnicity, sex, age, PMI, and ambient temperature) differences using an analysis of variance (ANOVA). Genus level assignments were examined for significant changes between genera, considering each genus separately. Here, OTU count data were analyzed using a generalized linear model with a negative binomial distribution^26^. Multivariate differences among groups (organ, manner of death, ethnicity, sex, age, PMI, and ambient temperature) were evaluated with Permutational Multivariate Analysis of Variance Using Distance Matrices function (ADONIS)^27^. For ADONIS, distances among samples first were calculated using unweighted or weighted UniFrac, and then an ANOVA-like simulation was conducted to test for group differences. Principal coordinates analyses and bar-plots were plotted to visualize relationships and differences between the groups (organ, manner of death, ethnicity, sex, age, PMI, and ambient temperature). Random Forest analyses were done to identify specific factors (i.e., sex, organ, OTUs) that significantly correlated with PMI using the Random Forest package and plots were done with standard commands in R^16^,^17^,^18^. Random forest analysis of the correlations between PMI and genera were conducted in R^28^, using the vegan^27^, DESeq2^26^, and phyloseq^29^ packages, and other plots were generated using the ggplot2 package^30^.

## Additional Information

**How to cite this article**: Javan, G. T. *et al*. Human Thanatomicrobiome Succession and Time Since Death. *Sci. Rep.* **6**, 29598; doi: 10.1038/srep29598 (2016).

## Supplementary Material

Supplementary Figure S1

Supplementary Table S1

Supplementary Table S2

## Figures and Tables

**Table 1:** Results of ADONIS based on unweighted and weighted UniFrac distances.

	Unweighted Unifrac						Weighted Unifrac					
	Df	Sum Sq.	Mean Sq.	F value	R2	Pr (*>*F)	Df	Sum Sq.	Mean Sq.	F value	R2	Pr (*>*F)
Organ	5	1.96	0.39	1.93	0.14	0.0010	5	2.33	0.47	3.71	0.22	0.0010
Age	1	0.14	0.14	0.71	0.01	0.8400	1	0.07	0.07	0.53	0.01	0.7820
Sex	1	0.34	0.34	1.70	0.02	0.0450	1	0.39	0.39	3.08	0.04	0.0210
PMI	1	0.38	0.38	1.90	0.03	0.0200	1	0.71	0.71	5.64	0.07	0.0020
Ambient Temp	1	0.23	0.23	1.15	0.02	0.2510	1	0.10	0.10	0.82	0.01	0.5060
Residuals	56	11.35	0.20		0.79		56	7.02	0.13		0.66	

**Table 2:** Results of ANOVA, testing for differences in the Chao1 richness and Shannon diversity among groups.

	Chao1 richness					Shannon diversity				
	Df	Sum Sq.	Mean Sq.	F value	Pr (*>*F)	Df	Sum Sq.	Mean Sq.	F value	Pr (*>*F)
Organ	5	23862.77	4772.55	0.52	0.7584	5	16.98	3.4	3.2	0.013
Age	1	2513.95	2513.95	0.28	0.602	1	0.25	0.25	0.24	0.6259
Sex	1	35056.52	35056.52	3.84	0.0551	1	16.45	16.45	15.51	0.0002
PMI	1	9052.64	9052.64	0.99	0.3239	1	1.55	1.55	1.47	0.2312
Ambient Temp	1	576.29	576.29	0.06	0.8026	1	0.04	0.04	0.04	0.8445
Residuals	56	511717.4	9137.81			56	59.38	1.06		
