# Supplementary material for: Human Thanatomicrobiome Succession and Time Since Death
Source: Sci Rep. 2016 Jul 14;6:29598. doi: 10.1038/srep29598 (PMC4944132; doi:10.1038/srep29598)
Supplement: Supplementary Figure S1 [file srep29598-s1.pdf]

Human Thanatomicrobiome Succession and Time Since Death

Gulnaz T. Javan[1*], Sheree J. Finley[2], Ismail Can[1], Jeremy E. Wilkinson[3], J. Delton Hanson[3], Aaron M. Tarone[4]

***Corresponding author:**

Gulnaz T. Javan

Email: gjavan@alasu.edu

Phone: +1 334-229-5202

Authors' affiliations:

[1] Forensic Science Program, Alabama State University, Montgomery, AL 36104

[2] Ph.D. Program in Microbiology, Alabama State University, Montgomery, AL 36104

[3] Research and Testing Laboratory, Lubbock, TX 79407

[4] Department of Entomology, Texas A&M University, College Station, TX, 77843

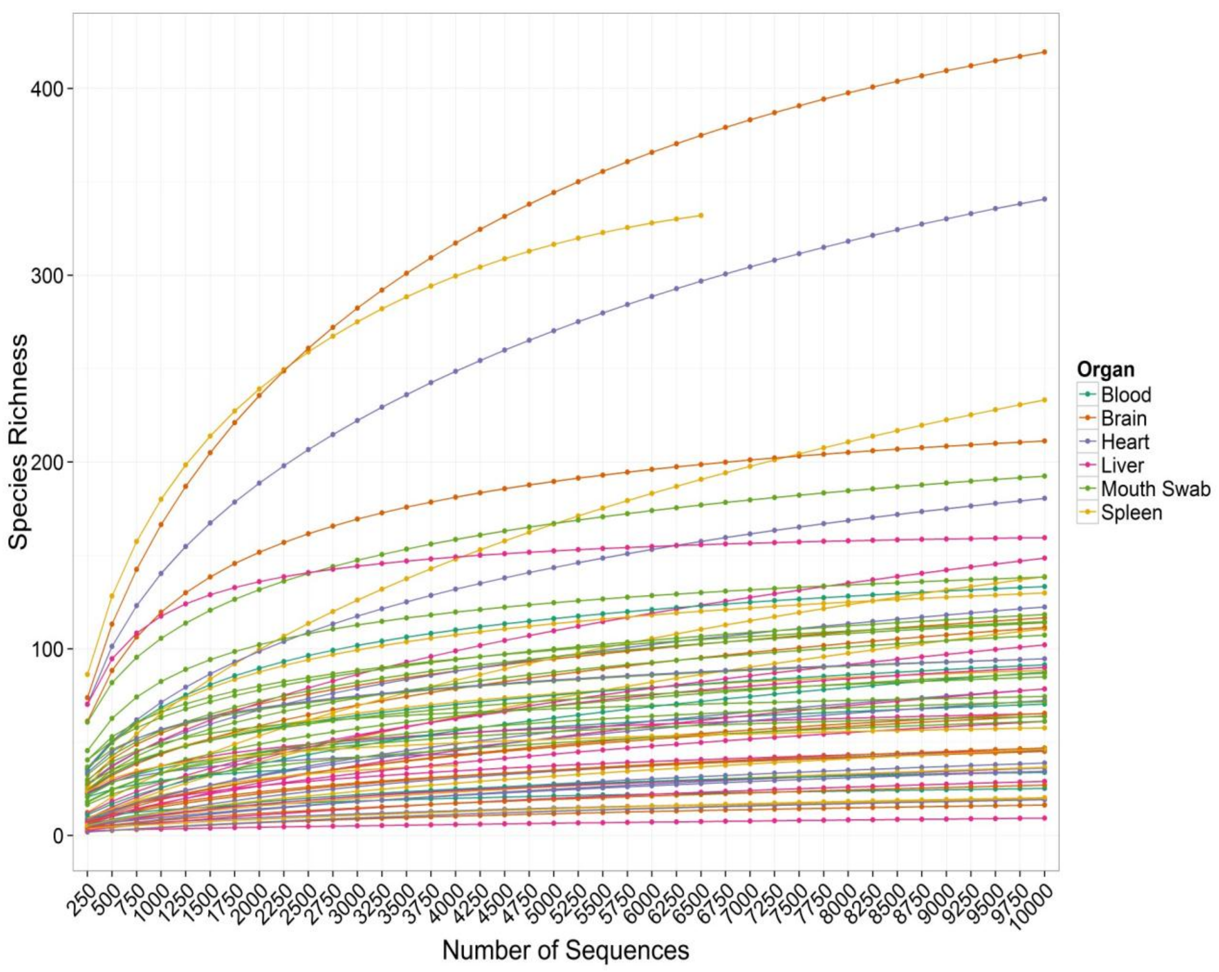

Figure S1. Rarefaction plot of the OTUs versus the number of sequences sampled per cadaver specimens. Subsampling was performed from 250 to 10000 reads in increments of 250 reads. The curves show that thanatomicrobiome species richness varies among different body samples.
